# Cardio-Vascular Extracellular Matrix: The Unmet Enigma

**DOI:** 10.3390/ijms27010544

**Published:** 2026-01-05

**Authors:** Ioannis Paraskevaidis, Elias Tsougos, Christos Kourek

**Affiliations:** 1 Medical School of Athens, National and Kapodistrian University of Athens, 15772 Athens, Greece; 2 Department of Cardiology, Hygeia Hospital, 15123 Athens, Greece; tsougos@yahoo.com

**Keywords:** heart failure, fibrosis, metalloproteinase, preserved ejection fraction, cardiovascular disease

## Abstract

The cardiac extracellular matrix (ECM) is a dynamic, tissue-specific scaffold essential for cardiovascular development, homeostasis, and disease. Once considered a passive structural framework, the ECM is now recognized as an active regulator of mechanical, electrical, and biochemical signaling in the heart. Its composition evolves from embryogenesis through adulthood, coordinating cardiomyocyte maturation, chamber formation, and postnatal remodeling. In pathological states, diverse stimuli—including ischemia, pressure or volume overload, metabolic dysfunction, and aging—disrupt ECM homeostasis, triggering fibroblast activation, myofibroblast transformation, and maladaptive collagen deposition. These processes underpin myocardial fibrosis, a key driver of impaired contractility, diastolic dysfunction, arrhythmogenesis, and heart failure across ischemic and non-ischemic cardiac diseases. ECM alterations also exhibit age- and sex-specific patterns that influence susceptibility to cardiovascular pathology. Advances in imaging and circulating biomarkers have improved fibrosis assessment, though limitations persist. Therapeutic strategies targeting ECM remodeling, including modulation of profibrotic signaling pathways, non-coding RNAs, cellular therapies, and nano-delivery systems, show promise but remain largely experimental. Collectively, expanding knowledge of ECM biology highlights its central role in cardiovascular physiology and pathology and underscores the need for targeted diagnostic and therapeutic innovations.

## 1. Introduction

Until a few years ago, it was believed that the extracellular matrix (ECM), a non-cellular tissue, functioned merely as a static cardiovascular scaffold without particular properties and with questionable utility. However, over time, its role and multifunctional characteristics have emerged as a significant area of research. Indeed, it has been documented that the ECM is an active, tissue-specific entity influenced by several biochemical, mechanical, and hemodynamic forces and involved in many functions of the cardiovascular system, including cell migration, cell differentiation, tissue growth, fibrosis, etc. [1,2,3,4]. It is part of the homeostatic process and plays an important role in the genesis and progression of several cardiovascular diseases, since structural components of the ECM are involved in linking proteins with growth factors, in cell receptor–binding properties, and thus in maintaining both vascular and myocardial function. Additionally, it participates in dead-cell replacement mechanisms in various cardiovascular pathologies, including ischemic and non-ischemic cardiac diseases such as myocardial infarction and cardiomyopathies [5].

Glycosaminoglycan (GAG) polymers such as hyaluronan (HA) and heparan sulfate are linked to proteoglycans and subsequently secreted. Sulphated proteoglycans (versican, aggrecan, etc.), forming aggregates with HA, have regulatory functions related to ECM hydration [6]. Heparan sulfate binds growth factors and cytokines, modifying their activity [7,8], whereas proteoglycan-link proteins promote interactions between ECM molecules and cellular receptors [9], including integrins. At the same time, members of the matrix metalloproteinase family (disintegrins, thrombospondins, etc.) maintain ECM equilibrium by regulating synthesis and degradation [10].

The cardiovascular ECM is not a static tissue; rather, it changes continuously throughout cardiovascular maturation and also after injury. After birth, ECM composition evolves in parallel with the cardiomyocyte transition from a regenerative to a reparative phenotype. Furthermore, collagen fibers, particularly collagen IV, and fibronectin interact with the myocardial cell basement membrane, supporting its structural organization. As a result, the ECM becomes more structural (with abundant collagen I, collagen III, and laminin) and less non-structural, adopting a porous, honeycomb-like architecture ready to support reparative processes through its reservoir of latent growth factors and cytokines. However, when the equilibrium between ECM synthesis and degradation is disrupted, deleterious effects occur, including sudden cardiac death [11]. At the initial stages, fibrosis may act as a protective or reparative response to harmful stimuli. When this process becomes prolonged, it transitions into excessive fibrosis characterized by fibroblast-to-myofibroblast transformation and heightened inflammatory activation, resulting in harmful mechanical and electrical consequences [12,13].

**Figure 1:**
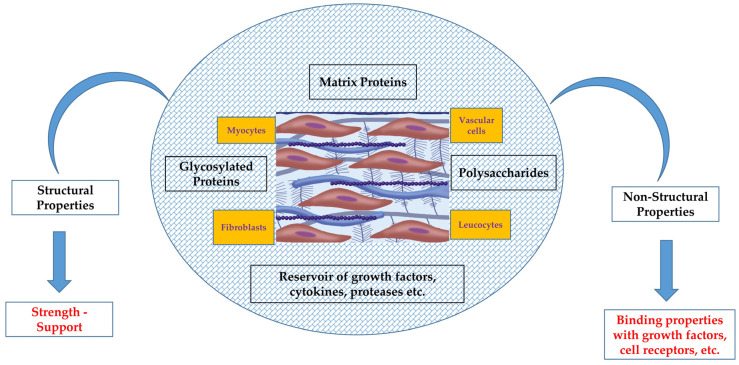
A fibrotic protein environment with structural and non-structural properties, myocytes, vascular cells, fibroblasts, leucocytes, etc., along with glycosylated proteins (proteoglycans), polysaccharides (glycosaminoglycans), and bioactive signaling molecules, forming the cardiac extracellular matrix network.

Taken together, these features highlight that the ECM is a living and dynamic tissue that interacts with cardiac mechanical and electrical properties, contributes to human homeostatic responses, and plays a key role in many cardiovascular diseases. It may therefore represent a promising field for the development of novel therapeutic strategies. Cardiac extracellular matrix (Figure 1), as an unresolved scientific enigma, unquestionably merits further investigation and research.

In this context, the primary objective of the present review is to provide an integrated and contemporary overview of the cardiac extracellular matrix as a dynamic, biologically active system that orchestrates myocardial structure, function, and disease across the lifespan. Beyond summarizing established mechanisms, we aim to synthesize emerging evidence on fibroblast heterogeneity, age- and sex-dependent extracellular matrix remodeling, and the shared fibrotic substrate underlying diverse heart failure phenotypes. By framing extracellular matrix remodeling as a unifying pathophysiological axis rather than a secondary epiphenomenon, this review seeks to distinguish itself from prior descriptive accounts and to highlight novel diagnostic and therapeutic implications relevant to precision cardiovascular medicine.

## 2. The Genesis and the Evolution of Cardiac Extracellular Matrix

Cardiac progenitor cells fuse during the embryonic phase, forming the primary heart tube, which consists of myocardial and endocardial layers separated by a thick ECM layer known as the cardiac jelly. The high–molecular–weight glycoprotein fibronectin binds to cell-surface receptors such as integrins and platelet-derived growth factor, forming complexes responsible for cell shape, division, and the developmental course of cardiomyocyte precursors [6]. Although the ECM does not directly regulate the morphological transformation from a simple tube to an elongated, looped structure, evidence suggests that when gelatinases such as MMP2 (zinc-dependent enzymes capable of cleaving ECM components) are inhibited, cells migrate from the heart tube toward more dorsal embryonic tissues, a process implicated in the development of torsional strain [14,15].

During the looping phase, when cardiac atria and ventricles form, ECM volume is reduced; however, even in smaller amounts, it retains a pivotal role in shaping cardiac chamber architecture. The trabecular configuration of the ventricles results from the equilibrium between ECM formation and degradation, the latter mediated primarily by metalloproteinases [16,17,18,19]. When this equilibrium is disrupted, various cardiac malformations may arise [6]. For example, when ECM functional properties are imbalanced, ventricular trabeculae persist abnormally, contributing to the development of non-compaction cardiomyopathy.

During fetal growth, fibroblasts infiltrate the myocardium, and connective tissue surrounds the myofilament network. If the normal sequence of events fails, construction of the cardiac scaffold becomes problematic. Accordingly, ECM constituents and integrin ligands “interact to coordinate the activity of cardiomyocyte mitogens secreted by fibroblasts” [6]. After birth, the ECM undergoes major changes that influence myocardial regenerative capacity and are accompanied by increased ventricular stiffness [20,21,22].

It is essential to recognize that the ECM is an integral component of the entire cardiovascular system and represents a critical factor that reacts and adapts to diverse stimuli. It influences synchronized myocardial contraction, passive ventricular expansion, overstretch prevention, and electrical conduction. It is responsible for valve coaptation, compression, and extension, and controls vessel elastic deformation and arterial contraction [6]. Under mechanical, neurohormonal, electrical, and other stimuli, ECM (through fibroblasts) adjusts its composition to respond appropriately. Following receptor signaling, through integrins, CD44, and others, multiple binding sites are activated, affecting fibroblast and cardiomyocyte behavior. When this coordinated interaction fails, various ischemic and non-ischemic cardiac diseases may emerge.

The ECM is an active, tissue-specific entity, displaying diverse characteristics across tissues and even within different regions of the same tissue. It is composed of fibrillogenesis structures, fibrillar and non-fibrillar collagen, proteoglycans, hyaluronan-binding proteins, all of which aggregate within the same microenvironment. Upon stimulation, the ECM can activate several receptors, including integrin heterodimers, discoidin domain receptors (collagen-sensitive), CD44 (which recognizes osteopontin), and associated enzymatic properties such as transglutaminases, lysyl oxidases, lysyl oxidase–like enzymes, lysyl hydroxylases, and tumor necrosis factor pathways [23,24,25].

Based on these observations, it is evident that the ECM is present from the beginning of life, undergoes continuous modification, and functions as a structural scaffold with important functional capacities, including intercellular communication and the incorporation of signaling molecules and mediators [26,27]. In the adult heart, cardiomyocytes are surrounded by ECM composed of collagen, laminin, perlecan, fibronectin, etc. These structures communicate through integrin receptors and provide essential functional properties such as migration, excitation, and intercellular communication. Collagen, the most abundant ECM component, provides necessary stiffness to the ventricular wall and microvasculature, supports resistance to external forces, and facilitates material exchange. It participates in myocardial contraction, relaxation, and electrical conduction [6,28].

Cardiac fibroblasts produce collagen as the first step toward fibrillogenesis, mainly collagen type I (85%) and type III (11%). These collagens drive ECM remodeling, allowing contractile forces to spread throughout the myocardial wall and enabling cardiomyocytes to exchange signals and maintain intercellular connectivity [26,29,30]. In medium and large blood vessels, the tunica media, located between the tunica intima and tunica adventitia, is composed of vascular smooth muscle cells, collagens, and elastin, and plays a major role in homeostatic adaptation to mechanical forces [30]. The tunica adventitia, in turn, consists of collagen, fibroblasts, and inflammatory cells [31]. Because of this blood vessel anatomic composition, elastic and viscous properties that facilitate continuous blood flow are preserved [32]. The altered ECM composition also affects arteries with small size; coronary arteries, which, for either genetic reasons or basement membrane proteins alteration, promote atherosclerosis and hence coronary artery disease [33].

## 3. Fibroblasts: The Masters of the Game

Myocytes, endothelial cells, smooth muscle cells, and fibroblasts are the main components of the cardiovascular system, with fibroblasts being the most abundant, comprising 40–60% of the total cell population [34]. Fibroblasts are present in every tissue; however, in the heart, they display a distinct morphology, appearing elongated rather than as flattened stellate cells, and demonstrating increased cellular activity. In cardiac tissue, they originate from the epicardium, infiltrate the cardiac walls, and produce the connective tissue that surrounds cardiomyocytes, forming the basis for ECM scaffold formation, which protects the heart from external forces and absorbs mechanical stress during contraction.

**Figure 2:**
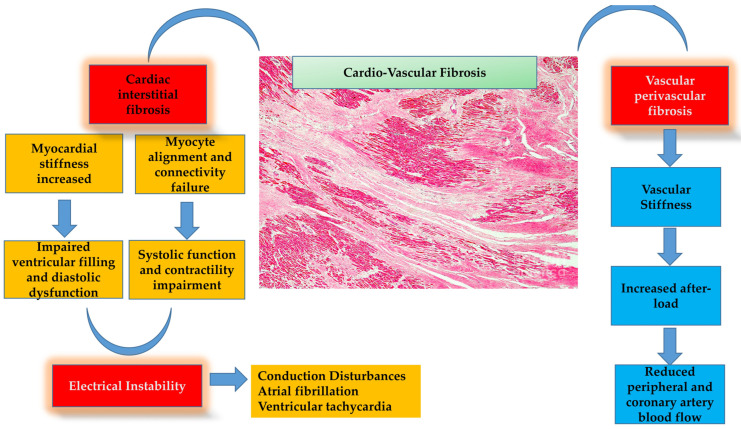
Cardiovascular fibrosis, when extensive after a severe injury, becomes detrimental, altering the anatomical and functional properties of the cardiovascular system. Deposition of collagen types I–III within both the cardiac interstitial space and the vascular perivascular space leads to myocardial stiffness and dysfunction, ultimately contributing to heart failure with either preserved or reduced ejection fraction.

Along with other ECM components, fibroblasts possess additional roles, including adhesion, growth, differentiation, proliferation, apoptosis, paracrine signaling, and homeostatic regulatory functions through continuous modeling and remodeling. They stimulate embryonic cardiomyocytes by promoting proliferation and adult cardiomyocytes by promoting hypertrophy. Following an insult, fibroblasts continue to grow and differentiate depending on the degree of myocardial damage (Figure 2) [34]. After a mechanical or chemical event, fibroblasts interact with cardiomyocytes and activate fibrocytes (fibroblast-derived cells from the bone marrow), which promote repair and immune responses.

Fibroblasts form a syncytium-like network connected through connexins [6,34], enabling intercellular communication. Although they lack classical electrophysiological properties, they contain potassium and sodium channels and have a negatively charged resting membrane potential. Under physiological conditions, collagen production, mainly types I and III, and collagen degradation are balanced and regulated by cytokines, growth factors, and metalloproteinases. When this equilibrium is disrupted, healing is altered, fibrosis increases, and matrix metabolism becomes dysfunctional.

Activation of cytokines by fibroblasts induces gene-expression changes that transform fibroblasts into myofibroblasts, which participate in inflammatory and reparative responses [34]. Myofibroblasts emerge following cardiac injury and have the capacity to anchor their internal microfilaments to extracellular fibronectin, thus creating a contractile mechanism. After injury, myofibroblasts spread throughout the heart, releasing cytokines and contributing to the inflammatory process. Although the precise mechanism governing this transformation is not fully understood, it appears to represent an adaptive response that increases adhesion proteins (paxillin, tensin, EDA-fibronectin, etc.) and stimulates the secretion of cytokines, particularly TGF-β and IL-1β. These, in turn, promote the transition from fibroblasts to myofibroblasts and enhance collagen production.

In addition, myofibroblasts affect ventricular gap junctions (Cx43), reduce intercellular communication, and significantly alter cardiac electrical properties [34]. As a result, ECM regulatory properties are lost, collagen deposition increases without adequate degradation, and fibrosis progresses. These changes lead to impaired mechanical and electrical cardiac function and ultimately to heart failure.

Fibroblasts are the predominant cell type in cardiac tissue because they produce collagens, proteoglycans, glycoproteins, matrix metalloproteinases, and their inhibitors, all essential for ECM remodeling.

### 3.1. Fibroblasts Activate a Cascade of Events

As mentioned before, fibroblasts are activated by biochemical, inflammatory, neuro-hormonal (interleukin-6, transforming growth factor-β, angiotensin II, endothelin-1, etc.), and mechanical factors (stress) [35,36]. Once activated, they release in abundance several inflammatory, angiogenetic, and other mediators in an effort to preserve cardiac tissue anatomy and function. They are also able to activate intracellular components (Smads protein family, mitogen-activated protein kinases, phosphoinositide-3-kinase/protein kinase B), altering gene expression and thereby promoting a pro-fibrotic cascade. Owing to these secretory and paracrine actions, they interact with adjacent myocardial cells (cardiomyocytes, endothelial cells, immune cells), thereby possessing multifunctional properties [37]. As expected, fibroblast activation marks the starting point of the fibrotic process, and when myocardial injury is severe, it drives the entire cascade toward scar formation, altering myocardial anatomical and functional properties (Figure 2).

It is noteworthy that fibroblasts appear at the injury site with different subpopulations demonstrating distinct actions. For instance, some fibroblast phenotypes exert anti-fibrotic effects, whereas others present more aggressive or harmful properties, promoting accelerated myocardial cell death [38], leading to adverse remodeling and arrhythmogenesis [39]. The fibrotic process, and consequently myocardial fibrosis, is observed regardless of the underlying etiology (hypertensive, diabetic cardiomyopathy, ischemic disease, etc.), promoting a cascade of deleterious effects (Figure 2). Initially, the process is beneficial, serving as a repair mechanism; however, over time, it becomes harmful, altering the anatomical and functional properties of the cardiovascular system, since collagen types I–III are deposited in both cardiac (interstitial) and vascular (perivascular) spaces [35]. Consequently, the mechanical and electrical functions of the cardiovascular system are reduced (and in more harmful cases severely altered), characterized by increased myocardial and vascular stiffness and diminished elastic properties. As a result, myocardial systolic and diastolic function, as well as electrophysiologic properties, are impaired, leading to heart failure [35,40].

It is worth noting that the degree and extent of fibrosis correlate linearly with sudden cardiac death; therefore, it may be an important topic for further investigation regarding potential new therapeutic approaches [41,42,43]. The activation of the fibrotic process is dynamic and represents a response to several stimuli, regardless of whether myocardial cell necrosis or apoptosis occurs [44]. Although its contribution to cardiovascular stiffness, myocardial connectivity, and electrophysiological instability is well established, the divergent role of fibroblast subpopulations remains to be elucidated [45,46,47]. Additionally, owing to the heterogeneous roles of fibroblasts, the fibrotic process is not easy to assess. Two types of fibrosis are recognized: (1) interstitial, either (a) reactive (during chronic stress or aging) or (b) infiltrative (interstitial accumulation of various substances, such as amyloidosis), and (2) tissue replacement with scar formation (following cell death). The first is diffuse and potentially reversible when the cause is removed, whereas the second is irreversible and characterized by cell death and scar formation. Nevertheless, cell death (either apoptosis or necrosis) can precede and accompany both types of interstitial fibrosis.

Regardless of the cause, the main fibroblast-activating factor is the severity of the injurious event. Activation depends on transforming growth factor-β (TGF-β), interleukin-6 (IL-6), angiotensin II, and endothelin-136 [48], which trigger intracellular signaling mechanisms and promote pro-fibrotic gene expression. Furthermore, inflammatory and angiogenetic mechanisms increase pro-inflammatory mediators that, along with cardiomyocyte and endothelial miscommunication, create a complex neighboring environment exhibiting harmful activities [37]. Conversely, as mentioned before, other fibroblast subtypes demonstrate opposite, protective effects. When this equilibrium is disrupted, homeostatic mechanisms fail, leading to increased cellular stress and death [35]. Consequently, the role of fibroblasts in extracellular matrix composition and function is complex and merits further attention to better understand and potentially identify more effective therapeutic approaches.

### 3.2. Fibroblast Heterogeneity Revealed by Contemporary High-Resolution Approaches

The concept of cardiac fibroblast heterogeneity has been substantially refined by contemporary high-resolution methodologies, particularly single-cell RNA sequencing, spatial transcriptomics, and genetic lineage-tracing approaches [35,49]. These techniques have overturned the traditional view of fibroblasts as a uniform population and instead revealed a spectrum of transcriptionally and functionally distinct fibroblast subtypes that dynamically evolve across development, injury, and disease [35,49].

Single-cell transcriptomic analyses have identified discrete fibroblast states in the adult and injured heart, including quiescent homeostatic fibroblasts, activated myofibroblasts, inflammatory fibroblasts, and stress-responsive secretory phenotypes [50,51]. Importantly, these populations exhibit context-dependent plasticity rather than fixed identities, transitioning between states in response to biomechanical stress, inflammatory cues, and neurohormonal signaling. Such findings provide a mechanistic basis for the variable fibrotic responses observed across different cardiac pathologies [50,51].

Genetic lineage-tracing studies have further clarified the cellular origins and fate of fibroblast subpopulations following myocardial injury [38,52]. These approaches demonstrate that resident cardiac fibroblasts, rather than circulating progenitors, constitute the principal source of myofibroblasts during scar formation, while also revealing phenotypically distinct fibroblast subsets that persist after injury and may contribute to long-term matrix homeostasis or adverse remodeling [35,48].

More recently, spatial transcriptomic analyses have added an additional layer of complexity by linking fibroblast transcriptional identity to anatomical localization [53]. Fibroblasts residing in the infarct core, border zone, perivascular regions, and atrial myocardium display distinct molecular signatures and functional programs, underscoring the importance of microenvironmental cues in shaping extracellular matrix remodeling [54]. Collectively, these data indicate that fibroblast heterogeneity is not merely descriptive but functionally consequential, with direct implications for myocardial mechanics, electrical stability, and the limited efficacy of non-selective antifibrotic therapies [35].

## 4. Extracellular Matrix Behavior in Cardiac Diseases

Myocardial fibrosis, the main anatomical characteristic of cardiac diseases regardless of the underlying cause, is defined as an excessive increase in collagen deposition within the interstitial space, leading to abnormal expansion of total myocardial tissue and distortion of cardiac architecture and function. Two major clinical entities are described in the context of cardiac diseases: ischemic and non-ischemic heart disease. These two conditions promote different phenotypic presentations of heart failure (reduced or preserved ejection fraction); therefore, it is of particular interest to explore the similarities and differences in the fibrotic process between them.

### 4.1. Extracellular Matrix in Non-Ischemic Heart Disease

Hypertensive heart disease, diabetic cardiomyopathy, hypertrophic cardiomyopathy, etc., are etiologic causes of non-ischemic heart failure, phenotypically expressed as heart failure with preserved ejection fraction. These conditions can lead to interstitial fibrosis of the reactive type (originating from the outer part of the ventricular free wall), characterized by thick collagen bands around cardiomyocytes and within the perivascular space of intramural arteries and arterioles. However, microscars are also present and represent the reparative type of fibrosis [55]. In any case, it is important to recognize the heterogeneity of fibrosis depending on the amount of collagen fiber accumulation, the type of collagen involved (collagen I–III), and the degree of intermolecular covalent linkage, all of which contribute to different clinical significance. For example, in hypertensive heart disease [56], low collagen deposition with normal covalent linkage represents a mild situation, whereas increased deposition with increased covalent linkage reflects a more severe one.

The reactive type of interstitial fibrosis results from various stimuli, including mechanical stress, metabolic alteration, endothelial inflammation, and sarcomere mutations [57,58,59]. This type of fibrosis represents the response that follows cell injury or death, activating damage-associated molecular patterns (immune activation), which in turn promote fibroblast proliferation and their transformation into secretory cells, the myofibroblasts. These cells possess autocrine and paracrine properties [60] and, depending on the degree of their activation, drive the fibrotic process (myofibroblast proliferation, altered metabolic status, etc.) to either a mild or severe response.

A cascade of events follows, starting with the secretion of procollagen type I amino-terminal proteinase, procollagen type I carboxy-terminal proteinase, and other enzymes that modify the synthesis of the procollagen precursor. Myofibroblasts also secrete other macromolecules such as osteopontin and fibronectin. Their secretory activity influences the fibrotic process by activating enzymes of the lysyl oxidase family, which catalyze and transform already formed collagen molecules into mature collagen fibers subsequently deposited in intercellular and perivascular spaces [61,62]. Are myofibroblasts the only source promoting interstitial fibrosis? Although essential, they appear not to be the sole contributors. M2 macrophages, lymphocytes, endothelial cells, estrogen receptors, androgen deficiency, etc., also participate in this process [61,62].

Of note, not all types of collagen deposition have the same functional consequence; the final clinical result depends on collagen type and its mechanical efficiency [63]. For example, collagen I produces a stiffer myocardium than collagen III, contributing to left ventricular diastolic dysfunction. However, in left ventricular systolic dysfunction (dilated cardiomyopathy), although the collagen I/collagen III ratio is increased, the left ventricle becomes both stiff and dilated because collagen functional capacity is reduced owing to cross-link instability and impaired force transmission [55,64,65]. Therefore, it remains questionable whether myocardial interstitial fibrosis is a unique characteristic of diastolic heart failure. Indeed, in patients with hypertensive heart disease, aortic stenosis, or diabetic cardiomyopathy, the amount of fibrosis is more severe in those with systolic dysfunction than in those with preserved ejection fraction [66,67,68]. This may suggest that heart failure with reduced or preserved ejection fraction shares the same underlying fibrotic pathophysiology, representing different expressions of the same disease [69]. In early HFpEF phenotypes, extracellular matrix expansion may therefore represent a primary driver of disease progression rather than a passive consequence of cardiomyocyte dysfunction.

### 4.2. Extracellular Matrix in Ischemic Heart Disease

#### 4.2.1. Acute Myocardial Ischemia

The term myocardial acute event includes both unstable angina and myocardial infarction. The primary difference between these two conditions lies in the extent of myocardial cell death. The extracellular matrix, and thus the healing response, reacts according to the degree of myocardial cellular injury. Indeed, the matrix response is linearly related to the severity and amount of myocardial cell death (tissue replacement type). Following myocardial infarction, the regenerative capacity of myocytes is limited. Because a substantial number of myocytes are lost and their regenerative ability is scarce, the homeostatic healing response is to fill the void with extracellular matrix components, leading to scar formation.

This filling process proceeds through three phases aimed at eliminating necrotic tissue and supporting structural repair: immune activation, proliferation, and maturation of matrix components. Endogenous inducers (myocardial cells, matrix molecules, etc.), along with activation of damage-associated molecular patterns, signal through receptors (Toll-like receptors) to activate immune mediators. These mediators then target affected myocardial and vascular tissues to restore structural integrity. At the same time, collagenases, gelatinases, and cathepsins become overactivated within the ischemic area, promoting reparative processes [70,71].

The first phase is followed by proliferation and maturation, which involve the activation of specialized matrix proteins (fibronectin, proteoglycans, etc.) and the participation of cardiomyocytes, fibroblasts and their transformation into myofibroblasts, vascular cells, etc., to regulate inflammation and control healing [72]. This process is not confined to the infarct region; rather, both immune and cardiac tissues are engaged. The non-infarcted myocardium responds to altered hemodynamics, and the bone marrow and spleen become activated, promoting the accumulation of leukocytes, mast cells, dendritic cells, etc. Healthy myocardium also contributes to collagen deposition toward the injured area, participating in the healing process through fibrosis [72].

This involvement aims to clear necrotic tissue (via phagocytosis, autophagy, degradation, etc.) and to limit fibrosis to the site of injury. Bioactive peptides (matrix proteases and intercellular proteins) enrich the intercellular space, regulate responsible growth factors, and help restrict fibrotic expansion [70]. Matrix metalloproteinases have multifunctional roles: they process tumor necrosis factor (and TNF-β), cytokines, interleukin-1β, and chemokines (CXCL12/stromal cell-derived factor, CCL2/monocyte chemoattractant protein), and interact with activated leukocytes, attempting to balance fibrosis and inflammation [73,74,75,76,77].

The reparative process must achieve two major goals: (1) preserve cardiac function as much as possible and (2) prevent myocardial wall rupture. To achieve the latter, macrophages, lymphocytes, vascular cells, growth factors, matricellular proteins, and others contribute, but the principal role is attributed to fibroblast/myofibroblast activity together with the plasmin system [70]. Additionally, non-fibrillar collagens (collagen IV), although not yet fully understood, appear to regulate ECM homeostasis by controlling infarct expansion and reducing adverse remodeling [78].

While myocardial infarction clearly triggers inflammatory and reparative responses leading to scar formation, the stimulus that terminates scar formation and halts the subsequent cascade remains unknown. For reasons still unclear, fibroblasts undergo apoptosis, activated myofibroblasts diminish, and growth factors and multicellular proteins decline, whereas phenotypically distinct fibroblasts may help maintain the fibrogenic, fibrinolytic equilibrium, preventing excessive scar formation [70]. As the scar matures, the newly formed vasculature regresses through platelet-derived growth factor activation and interactions between perivascular cells (pericytes) and endothelial cells [79,80].

#### 4.2.2. Chronic Myocardial Ischemia

In this setting, extracellular matrix remodeling is predominantly reparative and structural, with limited potential for reversal once mature scar architecture is established.

Patients with chronic myocardial ischemia exhibit high levels of collagens, metalloproteinases with elevated activity, and matricellular proteins [70,81,82]. However, it remains uncertain whether these extracellular matrix alterations result directly from chronic ischemia or reflect ECM responses to comorbid conditions (hypertension, diabetes, etc.). Experimental models, however, support a link between chronic ischemia and changes in ECM composition [83,84].

In patients with ischemic cardiomyopathy undergoing coronary artery bypass surgery or supported with a left ventricular assist device, matrix metalloproteinases (MMP-2, MMP-9, MMP-14) are highly activated, substantially modifying ECM composition and the myocardial environment [82,85,86,87]. Nonetheless, comorbidities such as pressure or volume overload and metabolic abnormalities influence ECM structure and thus myocardial function.

In pressure overload, various collagenases and gelatinases become activated, leading to an imbalance between fibrotic synthesis and degradation of ECM proteins, resulting in alterations in myocardial architecture and mechanical and electrical dysfunction [66,88,89,90,91,92]. In volume overload, the pathophysiologic sequence is not completely understood. Reduced collagen synthesis, together with high procollagen degradation driven by mechanical stress, inflammation (macrophages, mast cells, cytokines, etc.), and genetic alterations, has been proposed as a cause of cardiac dilation and remodeling [93,94,95,96,97].

Similarly, in individuals with metabolic disorders, free radical production, neurohumoral overexpression, adipokine secretion, and inflammation contribute to interstitial and perivascular fibrosis. In diabetic hearts, activation of transforming growth factor-β, along with macrophage, cardiomyocyte, and endothelial activation, promotes enhanced fibrogenic activity [59,98,99,100]. In obese patients, many of whom are also diabetic, increased microvascular inflammation further stimulates interstitial fibrosis. Additionally, a distinct obesity-related phenotype (linked to heart failure with preserved ejection fraction) has been identified, promoting further concentric cardiac hypertrophy [101].

### 4.3. Extracellular Matrix Remodeling in HFpEF and HFrEF: Causality, Timing, and Reversibility

An important unresolved question concerns whether extracellular matrix remodeling in heart failure represents a primary causal mechanism or a secondary adaptive response to myocardial injury. Available evidence suggests that extracellular matrix alterations function as both initiators and amplifiers of disease, depending on the underlying etiology and disease stage [102]. In conditions such as hypertensive heart disease, metabolic cardiomyopathy, and early HFpEF, diffuse interstitial fibrosis may precede overt cardiomyocyte dysfunction, contributing causally to increased myocardial stiffness, impaired relaxation, and microvascular dysfunction [103]. In this context, extracellular matrix remodeling is often reactive, progressive, and at least partially reversible when the inciting stimulus is removed or attenuated [104].

In contrast, in advanced HFrEF, particularly following extensive ischemic injury, extracellular matrix remodeling largely reflects a reparative response to irreversible cardiomyocyte loss [105,106]. Replacement fibrosis and mature scar formation restore structural integrity but impose permanent mechanical and electrical constraints, rendering extracellular matrix alterations predominantly secondary and largely irreversible [106,107]. Between these extremes lies a dynamic transitional phase in which maladaptive fibroblast activation and matrix expansion propagate myocardial dysfunction through mechano-electric uncoupling, inflammation, and impaired force transmission [108].

Accordingly, HFpEF and HFrEF may be best conceptualized not as distinct entities but as phenotypic expressions along a continuum of extracellular matrix remodeling, in which timing, severity, spatial distribution, and collagen cross-linking efficiency determine functional outcome and therapeutic responsiveness.

The diverse patterns of extracellular matrix remodeling across cardiac disease phenotypes are summarized in Table 1.

## 5. Extracellular Matrix in Aging and Sex Characteristics

### 5.1. Aging

Aging affects cardiac diastolic properties because of key changes in extracellular matrix architecture and composition, as well as in cardiac myocytes, which become hypertrophic. A large amount of extracellular matrix accumulates as fibroblasts transform into activated myofibroblasts, with collagen deposition (mainly collagen types V and VI) altering cell-to-cell communication, cellular orientation, and promoting fiber disarray, ultimately leading to myocardial functional depression [109,110,111,112]. Although it remains questionable whether aging should be defined as a pathological entity or a normal biological process, it is characterized by a decline in homeostatic properties that, to some extent, represents the expected sequence of life. The etiologic explanation for this decline is not yet fully understood.

Several mechanisms have been proposed, suggesting an imbalance between detrimental influences and protective pathways. DNA damage, epigenetic changes, telomere shortening, nutritional and metabolic abnormalities, mitochondrial dysfunction, and advanced glycation end-products may exceed the capacity of impaired protective mechanisms such as autophagy, clearance of unfolded proteins, or chronic inflammation control. Importantly, senescence-associated secretory phenotype activity becomes impaired, altering the recruitment of immune cells responsible for cell clearance [113] and affecting mediators, fibrotic and pro-hypertrophic factors, homeostatic regulators, and matrix metalloproteinases [114]. Regardless of the proposed etiologies, the outcome indicates that aging is associated with increased collagen I/VI, vitronectin, and fibronectin and a concomitant reduction in fibulin-5, resulting in altered cardiac composition and architecture [115,116].

It is worth mentioning that it remains unclear whether cardiac chambers age in a similar manner. Experimental models based on transcriptomic data suggest distinct aging patterns between the right and left ventricles, possibly due to differences in gene-dependent metabolic pathways [117].

### 5.2. Sex Characteristics

Several studies have demonstrated the effect of biological sex on aging and consequently on extracellular matrix composition and cardiovascular disease [114,116,118,119]. Estrogens exert well-established protective effects, including anti-fibrotic, anti-apoptotic, antioxidative, and anti-inflammatory properties, and these actions are influenced by chromosomal composition [114,118,120]. Conversely, reduction or loss of estrogens facilitates collagen accumulation, oxidative stress, inflammation, and endothelial dysfunction [121], effects similar to those associated with testosterone, which predisposes to cardiac hypertrophy, collagen activation, and fibrosis [114,118,122]. The protective effect of estrogens diminishes after menopause, underscoring their pivotal role [122]. This is because genes regulating collagen deposition and inflammatory responses become suppressed, leading to excessive collagen accumulation and initiation of inflammatory cascades [123].

Interestingly, after gonadectomy, cardiac scar formation appears chromosome-dependent, making females more vulnerable or susceptible. Thus, both female chromosomes and hormones provide protective roles, and their suppression initiates a male-like extracellular matrix response [124]. Likewise, valvular interstitial cells exhibit sex-dependent gene expression that modulates fibroblast-to-myofibroblast transformation and alters metalloproteinase behavior, thereby promoting fibrosis [125,126]. Accordingly, the above concepts reinforce and expand our understanding of sex-related differences in extracellular matrix behavior.

## 6. Diagnostic Procedures

The detection of myocardial fibrosis, regardless of type, interstitial or replacement, is a complex issue, and significant progress is still required, demanding further research. Currently, two major approaches exist: invasive and non-invasive methods. Non-invasive procedures include biomarker assessment through blood samples and imaging techniques. From a clinical standpoint, the assessment of myocardial fibrosis and extracellular matrix remodeling remains characterized by a substantial translational gap between experimental advances and routine practice. While numerous invasive, circulating, and imaging-based techniques have demonstrated mechanistic or prognostic value in research settings, only a limited subset is currently applicable, validated, and actionable in everyday clinical care. The following sections therefore distinguish between diagnostic approaches that are established in clinical practice and those that remain primarily investigational.

### 6.1. Invasive Techniques

Although considered the gold standard, the invasive approach has several inherent limitations. It is performed via endomyocardial biopsy followed by histopathological analysis, an obviously unpleasant and not risk-free procedure. Moreover, sample acquisition may fail, either because fibrotic areas are missed or because it is impossible to explore the entire myocardium comprehensively.

### 6.2. Biomarkers

During the fibrotic phase, fibroblasts and myofibroblasts secrete structural and non-structural proteins that may be detected in the bloodstream. Procollagens (types I–III), elastin, fibronectin [127,128,129], and fibrotic mediators such as galectin-3 and soluble suppression of tumorigenicity-2 (sST2) can be considered biomarkers of myocardial fibrosis [130,131]. However, both procollagen type I carboxy-terminal propeptide and type III procollagen peptide, despite their promising role, are not widely used in clinical practice because of low cardiac specificity. Galectin-3 and sST2, although recommended for risk stratification [132], also lack adequate cardiac specificity [133]. Likewise, although microRNAs (miR-29b, miR-203, miR-4454, miR-133a, etc.) regulate fibrosis, their clinical utility has not yet been robustly established [129].

Non-invasive imaging modalities include echocardiography, nuclear imaging, cardiac computed tomography, and cardiovascular magnetic resonance imaging.

Despite their prognostic utility, most circulating fibrosis-related biomarkers currently serve as adjunctive risk stratification tools rather than definitive diagnostic markers of myocardial extracellular matrix remodeling in routine clinical practice.

### 6.3. Non-Invasive Imaging Techniques

As far as echocardiographyis concerned, several attempts have been made to indirectly identify myocardial fibrosis [134], using longitudinal strain, mechanical dispersion, myocardial work index, etc., but echocardiography is highly operator-dependent and limited by acoustic windows, restricting its reliability.

Single-photon emission computed tomography and positron emission tomography have been evaluated for fibrosis detection [129,135]; however, their accuracy in clinical human populations remains to be confirmed.

Computed tomography (CT) has been proposed as an alternative means of assessing extracellular volume as an imaging biomarker, but limitations, such as high heterogeneity, limited multicenter validation, and various confounding factorsl significantly affect reproducibility [136].

Cardiovascular magnetic resonance (CMR) is currently the primary non-invasive tool for detecting myocardial fibrosis [137] through late gadolinium enhancement (LGE), myocardial strain assessment, and T1-mapping techniques. Its value has been demonstrated in both experimental [138] and human studies [139,140,141]. Nonetheless, despite being the most promising method, certain limitations, field strength variability, segmentation techniques, low spatial sensitivity, heart rate variability, etc., remain challenges that must be overcome [142,143].

Among non-invasive modalities, cardiovascular magnetic resonance with late gadolinium enhancement and T1-mapping represents the most clinically established approach for fibrosis assessment, whereas emerging techniques such as CT-derived extracellular volume quantification and advanced nuclear imaging remain largely confined to specialized centers and research protocols [144].

Overall, the clinical translation of extracellular matrix–focused diagnostics is currently limited by issues of tissue specificity, standardization, accessibility, and cost. While advanced biomarkers, imaging metrics, and molecular signatures offer valuable mechanistic insights and hold promise for precision phenotyping, their routine implementation requires further validation, harmonization of acquisition protocols, and demonstration of incremental clinical benefit. Bridging this gap will be essential for transforming extracellular matrix assessment from a research tool into a clinically actionable component of cardiovascular care.

## 7. Therapeutic Approaches

The extracellular matrix, as previously discussed, has multifunctional properties that influence both cardiac mechanical and electrical activity and plays a fundamental role in cardiac homeostatic equilibrium. However, although there is a clear need to identify therapeutic targets capable of restoring post-injury homeostatic dysfunction, this remains an unmet challenge, likely because fibroblasts demonstrate significant spatial and functional heterogeneity. Therefore, finding a unified therapeutic approach is difficult, making it reasonable to focus greater attention on fibroblast heterogeneity and their sub-phenotypic characteristics. Fibroblasts residing in the atria contribute differently to cardiac function than those found in the ventricles or around cardiac vessels. In fact, atrial fibroblasts regulate electrical properties, ventricular fibroblasts exert mechanical effects via structural extracellular matrix secretion, and vascular fibroblasts promote angiogenic integrity [35,50,145]. Interestingly, they respond not only under physiological conditions but also after various insults or diseases, exhibiting high specificity according to their anatomical location and phenotype [146,147].

Fibroblasts can be categorized phenotypically into quiescent, myofibroblast-producing, inflammatory, and secretory types. Given their heterogeneity, both in location and phenotype, and the fact that fibroblasts transform from one phenotype to another following injury, it becomes clear that no single therapeutic strategy can be universally applied. In this context, several experimental studies have targeted profibrotic fibroblasts, yet none have produced robust or consistent outcomes [35,148,149]. The failure of uniform antifibrotic strategies may, at least in part, reflect the profound spatial and functional heterogeneity of cardiac fibroblast subpopulations revealed by single-cell and lineage-tracing studies.

Additionally, various signaling pathways have been proposed as therapeutic targets. For example, inhibition of the TGF-β pathway has produced ambiguous results, while modulation of the Wnt/β-catenin pathway [150] and notch signaling [151] has faced scientific and technical limitations. Other potential antifibrotic strategies have explored histone deacetylase inhibition [152,153,154] and the use of non-coding RNAs [155,156,157,158,159]. More recent approaches, including cell-based therapies [160,161,162] and nano-drug delivery systems [163,164], have emerged with promising findings, offering a pathway toward precision-medicine–based therapeutic models.

### 7.1. Lessons from Unsuccessful Antifibrotic Strategies: The Challenge of Fibroblast Heterogeneity

Despite extensive preclinical and translational efforts, most antifibrotic strategies targeting extracellular matrix remodeling have failed to demonstrate consistent clinical benefit [165]. This translational gap likely reflects an oversimplified conceptualization of cardiac fibrosis as a uniform, static process, rather than a dynamic, spatially and temporally regulated response driven by heterogeneous fibroblast populations.

Many therapeutic approaches have focused on broadly inhibiting profibrotic signaling pathways, such as transforming growth factor-β, angiotensin II, or downstream matrix synthesis enzymes [166,167]. While effective in reducing collagen deposition in experimental models, these strategies often interfere with essential reparative and homeostatic functions of fibroblasts, leading to limited efficacy or unacceptable adverse effects in clinical settings [166,167]. Such outcomes underscore the dual role of fibroblasts as both mediators of pathological fibrosis and indispensable regulators of myocardial integrity.

Emerging evidence from single-cell and lineage-tracing studies indicates that fibroblasts exist in multiple, functionally distinct states that vary according to anatomical location, disease stage, and microenvironmental cues [168,169]. Non-selective antifibrotic interventions are therefore unlikely to uniformly suppress maladaptive fibroblast activity while preserving beneficial reparative phenotypes [168,169]. In addition, fibroblast plasticity allows cells to transition between phenotypic states, further complicating static therapeutic targeting [170].

These insights suggest that the failure of prior antifibrotic therapies may stem less from an invalid biological target and more from insufficient therapeutic precision. Future strategies will likely require temporally and spatially restricted modulation of specific fibroblast subpopulations or signaling programs, ideally guided by molecular phenotyping and disease stage. Such an approach may reconcile the need to limit excessive fibrosis while preserving the adaptive and structural functions of the extracellular matrix.

### 7.2. Pharmacological Modulation of the Cardiovascular Extracellular Matrix Across Cardiac Disease Phenotypes

Beyond experimental strategies, several pharmacological agents currently used in cardiovascular medicine have been reported to influence extracellular matrix remodeling across different cardiac disease phenotypes. Importantly, the magnitude, mechanism, and reversibility of these effects appear to be disease- and stage-dependent.

In HFpEF, diffuse interstitial fibrosis and collagen cross-linking are central pathophysiological features [171]. Direct antifibrotic intervention with pirfenidone has demonstrated reduction in myocardial fibrosis as assessed by cardiovascular magnetic resonance, providing proof-of-concept that ECM remodeling may be at least partially reversible in selected HFpEF populations [172,173]. In addition, SGLT2 inhibitors and sacubitril/valsartan have been associated with favorable changes in fibrosis-related biomarkers and extracellular volume, likely through indirect modulation of fibroblast activation, inflammation, and myocardial loading conditions [174,175,176]. Nevertheless, these effects remain modest and are most evident in early or less advanced disease.

In HFrEF, ECM remodeling largely reflects a combination of diffuse interstitial fibrosis and irreversible replacement fibrosis following cardiomyocyte loss [105]. Standard guideline-directed therapies, including renin–angiotensin–aldosterone system inhibitors, β-blockers, mineralocorticoid receptor antagonists, and ARNI therapy, have been shown to attenuate adverse ECM turnover, primarily by reducing profibrotic signaling rather than reversing established scar tissue [177,178]. Thus, in HFrEF, ECM modulation appears predominantly secondary and supportive, with limited reversibility once advanced remodeling is established.

After myocardial infarction, ECM-directed interventions face the dual challenge of preserving essential scar formation while limiting excessive or maladaptive fibrosis [179]. Experimental and early translational studies suggest that modulation of matrix metalloproteinases, TGF-β signaling, and collagen cross-linking enzymes can influence infarct expansion and remote myocardial remodeling [88,180]. However, broad inhibition of these pathways has frequently failed clinically, underscoring the necessity of temporally and spatially restricted ECM modulation rather than indiscriminate antifibrotic suppression.

In pressure-overload states, reactive interstitial fibrosis may precede overt systolic dysfunction [103,181]. Antihypertensive therapies, particularly RAAS inhibitors and mineralocorticoid receptor antagonists, have demonstrated partial regression of myocardial fibrosis, especially when instituted early [181]. These observations support a causal role of ECM remodeling in disease progression and suggest a therapeutic window during which ECM alterations remain modifiable.

Diabetic cardiomyopathy is characterized by inflammatory and metabolically driven ECM expansion [182,183]. SGLT2 inhibitors, RAAS blockade, and improved metabolic control have been associated with reduced fibrotic signaling and ECM accumulation in both experimental and clinical studies [184,185,186]. However, advanced glycation end-product–mediated collagen cross-linking likely limits reversibility in long-standing disease.

Collectively, these observations indicate that while pharmacological modulation of the cardiovascular ECM is feasible, therapeutic success depends on disease context, timing, fibroblast phenotype, and the balance between adaptive and maladaptive remodeling.

A summary of compounds influencing extracellular matrix remodeling across cardiac disease phenotypes is provided in Table 2.

## 8. Conclusions

The cardiac extracellular matrix is not a passive structural bystander but a highly dynamic, tissue-specific system that actively regulates myocardial mechanics, electrical stability, and cellular communication. Across development, aging, and disease, extracellular matrix remodeling emerges as a central integrator of biomechanical stress, inflammatory signaling, and neurohormonal activation.

A key concept highlighted in this review is that myocardial fibrosis represents a shared biological substrate across ischemic and non-ischemic heart disease, as well as across heart failure phenotypes traditionally classified by ejection fraction. Differences in clinical presentation appear to reflect variations in extracellular matrix composition, spatial distribution, and cross-linking efficiency rather than fundamentally distinct disease entities. Furthermore, accumulating evidence underscores the importance of fibroblast heterogeneity and context-specific extracellular matrix responses, influenced by anatomical location, aging, and biological sex. These factors likely explain the limited success of uniform antifibrotic strategies and argue for a more nuanced, precision-based therapeutic approach. Finally, a critical lesson from prior therapeutic failures is that effective modulation of cardiac fibrosis will require targeting specific fibroblast states and remodeling phases rather than indiscriminate suppression of extracellular matrix production.

Advancing our understanding of extracellular matrix biology, particularly its dynamic regulation, cellular diversity, and interaction with systemic factors, will be essential for the development of more accurate diagnostic tools and targeted interventions. Future research should therefore move beyond collagen quantification alone and focus on extracellular matrix functionality as a modifiable determinant of cardiovascular disease progression.

## Figures and Tables

**Table 1:** Summary of extracellular matrix remodeling patterns across major cardiac diseases, highlighting fibrosis type, fibroblast involvement, functional consequences, and potential reversibility.

Cardiac Condition	Dominant ECM Remodeling Type	Fibrosis Pattern	Key Fibroblast Features	Functional Consequences	Reversibility Potential
Hypertensive heart disease	Reactive interstitial fibrosis	Diffuse interstitial and perivascular	Activated fibroblasts, moderate myofibroblast differentiation	Increased myocardial stiffness, diastolic dysfunction	Partial (early stages)
Diabetic cardiomyopathy	Reactive + inflammatory fibrosis	Interstitial and perivascular	Pro-inflammatory fibroblast phenotypes, macrophage interaction	Diastolic dysfunction, microvascular impairment	Limited–partial
HFpEF	Diffuse interstitial fibrosis	Non-replacement, collagen cross-linking	Heterogeneous fibroblast activation	Impaired relaxation, increased LV stiffness	Potentially reversible (early)
Acute myocardial infarction	Replacement fibrosis	Localized scar formation	Myofibroblast-dominated	Structural stabilization, arrhythmogenic substrate	Irreversible
Ischemic cardiomyopathy (HFrEF)	Replacement + diffuse remodeling	Scar + remote interstitial fibrosis	Persistent activated fibroblasts	Systolic dysfunction, dilation	Largely irreversible
Pressure overload (e.g., aortic stenosis)	Reactive fibrosis	Diffuse interstitial	Mechanosensitive fibroblasts	Diastolic ± systolic dysfunction	Partial (post-unloading)
Volume overload (e.g., mitral regurgitation)	ECM degradation-dominant	Reduced collagen integrity	Hypofibrotic fibroblast phenotype	Ventricular dilation	Poorly reversible
Aging-related remodeling	Progressive interstitial fibrosis	Diffuse, collagen I/VI accumulation	Senescent fibroblasts	Increased stiffness, reduced reserve	Limited
Infiltrative cardiomyopathies (e.g., amyloidosis)	Infiltrative fibrosis	ECM expansion by deposited material	Secondary fibroblast activation	Restrictive physiology	Disease-dependent

ECM, extracellular matrix; HFpEF, heart failure with preserved ejection fraction; HFrEF, heart failure with reduced ejection fraction; LV, left ventricle.

**Table 2:** Pharmacological compounds influencing extracellular matrix remodeling across cardiac diseases.

Disease Context	Representative Compounds	Primary ECM-Related Effects	Evidence Level	Reversibility
HFpEF	Pirfenidone; SGLT2 inhibitors; ARNI	Reduced interstitial fibrosis, ↓ ECV, ↓ profibrotic signaling	Clinical (CMR, biomarkers)	Partial (early disease)
HFrEF	ACEi/ARB; MRA; ARNI	Attenuation of ECM turnover, ↓ collagen synthesis	Large RCTs (indirect ECM endpoints)	Limited
Post-MI remodeling	RAAS blockade; experimental MMP/TGF-β modulation	Controlled scar formation, ↓ adverse remodeling	Preclinical + early translational	Stage-dependent
Hypertensive heart disease	RAAS inhibitors; MRA	Regression of reactive fibrosis	Clinical	Partial
Diabetic cardiomyopathy	SGLT2 inhibitors; RAAS blockade	↓ inflammatory and metabolic fibrotic signaling	Clinical + experimental	Limited–partial

ACEi, angiotensin-converting enzyme inhibitor; ARB, angiotensin II receptor blocker; ARNI, angiotensin receptor–neprilysin inhibitor; ECM, extracellular matrix; ECV, extracellular volume; HFpEF, heart failure with preserved ejection fraction; HFrEF, heart failure with reduced ejection fraction; MI, myocardial infarction; MMP, matrix metalloproteinase; MRA, mineralocorticoid receptor antagonist; RAAS, renin–angiotensin–aldosterone system; SGLT2, sodium–glucose cotransporter 2; TGF-β, transforming growth factor beta; ↓, decrease.

## Data Availability

No new data were created or analyzed in this study.

## Abbreviations

The following abbreviations are used in this manuscript:

CMR	Cardiac Magnetic Resonance
CT	Computed Tomography
CXCL	C-X-C Motif Chemokine Ligand
ECM	Extracellular Matrix
ECV	Extracellular Volume
FAP	Fibroblast Activation Protein
GAL-3	Galectin-3
HFpEF	Heart Failure with Preserved Ejection Fraction
HFrEF	Heart Failure with Reduced Ejection Fraction
LGE	Late Gadolinium Enhancement
MMP	Matrix Metalloproteinase
PDGF	Platelet-Derived Growth Factor
PG	Proteoglycan
PICP	Procollagen Type I Carboxy-Terminal Propeptide
PIIINP	Procollagen Type III N-Terminal Propeptide
ROS	Reactive Oxygen Species
sSt2	Soluble Suppression of Tumorigenicity-2
TGF-β	Transforming Growth Factor-β
TIMP	Tissue Inhibitor of Metalloproteinases
